# Relative contributions of preprandial and postprandial glucose exposures, glycemic variability, and non-glycemic factors to HbA_**1c**_ in individuals with and without diabetes

**DOI:** 10.1038/s41387-018-0047-8

**Published:** 2018-06-01

**Authors:** Kristine Færch, Marjan Alssema, David J. Mela, Rikke Borg, Dorte Vistisen

**Affiliations:** 1 Steno Diabetes Center Copenhagen, Gentofte, Denmark; 2 Unilever Research and Development Vlaardingen, Vlaardingen, The Netherlands; 3 Department of Epidemiology and Biostatistics, Amsterdam Public Health Research Institute, VU University Medical Center, Amsterdam, The Netherlands; 4 Zealand University Hospital, Roskilde, Denmark

## Abstract

**Background/objective:**

There is substantial interest in dietary approaches to reducing postprandial glucose (PPG) responses, but the quantitative contribution of PPG to longer-term glycemic control (reflected in glycated hemoglobin, HbA_1c_) in the general population is not known. This study quantified the associations of preprandial glucose exposure, PPG exposure, and glycemic variability with HbA_1c_ and estimated the explained variance in HbA_1c_ in individuals with and without type 2 diabetes (T2D).

**Subjects/methods:**

Participants in the A1c-Derived Average Glucose (ADAG) study without T2D (*n* = 77) or with non-insulin-treated T2D and HbA_1c_<6.5% (T2D_HbA1c < 6.5%_, *n* = 63) or HbA_1c_ ≥ 6.5% (T2D_HbA1c ≥ 6.5%_, *n* = 34) were included in this analysis. Indices of preprandial glucose, PPG, and glycemic variability were calculated from continuous glucose monitoring during four periods over 12 weeks prior to HbA_1c_ measurement. In linear regression models, we estimated the associations of the glycemic exposures with HbA_1c_ and calculated the proportion of variance in HbA_1c_ explained by glycemic and non-glycemic factors (age, sex, body mass index, and ethnicity).

**Results:**

The factors in the analysis explained 35% of the variance in HbA_1c_ in non-diabetic individuals, 49% in T2D_HbA1c < 6.5%_, and 78% in T2D_HbA1c ≥ 6.5%_. In non-diabetic individuals PPG exposure was associated with HbA_1c_ in confounder-adjusted analyses (*P* < 0.05). In the T2D_HbA1c < 6.5%_ group, all glycemic measures were associated with HbA_1c_ (*P* < 0.05); preprandial glucose and PPG accounted for 14 and 18%, respectively, of the explained variation. In T2D_HbA1c ≥ 6.5%_, these glycemic exposures accounted for more than 50% of the variation in HbA_1c_ and with equal relative contributions.

**Conclusions:**

Among the glycemic exposures, PPG exposure was most strongly predictive of HbA_1c_ in non-diabetic individuals, suggesting that interventions targeting lowering of the PPG response may be beneficial for long-term glycemic maintenance. In T2D, preprandial glucose and PPG exposure contributed equally to HbA_1c_.

## Introduction

Hemoglobin A_1c_ (HbA_1c_) reflects glycemic exposure in the previous 8–12 weeks^1^. The level of HbA_1c_ is in addition to glycemia determined by the lifespan of erythrocytes, which is affected by nutritional deficiencies, for example, iron deficiency anemia and vitamin B_12_ deficiency^2^. In addition to nutritional deficiencies sex, genetic factors, and hematologic parameters are non-glycemic factors affecting HbA_1c_ concentrations^1^.

High HbA_1c_ concentrations are associated with an increased risk for cardiovascular disease and mortality in individuals with and without type 2 diabetes (T2D)^3–6^. Individuals without T2D spend a considerable amount of time during the day at glucose concentrations considered to be prediabetic (>7.8 mmol/L)^7^. Accordingly, lifestyle or pharmaceutical interventions targeting lowering of postprandial glucose (PPG) directly or via the underlying insulin sensitivity are relevant even in the non-diabetic population^8^. However, the exact contribution of normally experienced, daily PPG exposures and daytime glucose variability to variation in HbA_1c_ in individuals with and without T2D is currently unknown, because few studies have captured these fluctuations over sustained periods under free-living conditions, for example, by use of continuous glucose monitoring (CGM)^8, 9^.

In the current analysis, we aimed to compare the strength of the associations of real-life preprandial and PPG exposures as well as glycemic variability with HbA_1c_ concentrations in non-diabetic individuals and in persons with non-insulin-treated T2D with different levels of glycemic control. Moreover, we aimed to estimate the variance in HbA_1c_ concentrations explained by preprandial and PPG exposures, glycemic variability, and non-glycemic characteristics in these subgroups.

## Materials/subjects and methods

### Study population

The A1c-Derived Average Glucose (ADAG) study is a multicenter study including 11 centers in the United States, Europe, Africa, and Asia. From January 2006 to March 2008, 268 individuals with T1D, 159 with T2D, and 80 free of diabetes completed a 16-week period of intensive CGM and self-monitoring of blood glucose (SMBG). Individuals without diabetes had no family history of diabetes and a fasting plasma glucose concentration ≤5.4 mmol/L after an overnight fast. Persons with diabetes had to have stable glycemic control as evidenced by two HbA_1c_ values within 1 percentage point (~11 mmol/mol) of each other in the 6 months before recruitment. Diabetes management was left to the participants and their usual health care providers. Individuals with T1D were not considered in this study because we did not want to include individuals on insulin treatment. We further excluded 60 persons with T2D who were treated with insulin and five because of missing BMI (two with diabetes and three without), leaving 77 individuals without diabetes (non-DM) and 97 participants with T2D for the present analysis. Participants with T2D were further subdivided into those meeting and exceeding the target HbA_1c_ level for diabetes management of <6.5%/48 mmol/mol (respectively “T2D_HbA1c<6.5%_,” *n* = 63; and “T2D_HbA1c≥6.5%_,” *n* = 34).

The ADAG study was approved by the human studies committees for each of the participating institutions. Written informed consent was obtained from all participants before inclusion in the study.

### Data collection and study procedures

#### Characteristics

Clinical data collected at baseline included age, sex, height, weight, waist circumference, blood pressure, and treatment with insulin, lipid-lowering, or antihypertensive medication. Blood samples for HbA_1c_ and plasma lipids were obtained at baseline and monthly for 3 months (i.e., four repeat measurements in total). Information on ethnicity was coded as being of white or non-white origin.

#### Continuous glucose monitoring

Measures of glycemia were performed using the blinded CGM system (Medtronic Minimed, Northridge, CA, USA), which measures glucose concentrations every 5 min. The measurements were performed for at least 2 days at baseline and then again for at least 2 days every 4 weeks during the next 12 weeks. At least one successful 24-h profile out of the 2–3 days of monitoring with no gaps >120 min and a mean absolute difference of <18% compared with the Hemocue calibration results was required to be included in the analysis. Measures from the first 2 h of CGM measurement were excluded, since this period is considered an unstable calibration period. During the CGM measurement periods, the participants performed daily 8-point SMBG profiles with a HemoCue meter (HemoCue Glucose 201 Plus, Hemocue, Ängelholm, Sweden). SMBG was performed right before and 90 min after breakfast, lunch, and dinner as well as at bedtime and at 3 a.m. The SMBG measurements were used for calibration purposes, and the time points of SMBG measurements were used for definition of preprandial and postprandial periods.

#### Laboratory analyses

Blood samples taken at the clinical examinations were frozen at −80 °C and were sent on dry ice by overnight shipment to a central laboratory. As the ADAG study was part of the standardization of HbA_1c_ measurements, samples for determination of HbA_1c_ were analyzed with four different DCCT-aligned assays, including a high-performance liquid chromatography assay (Tosoh G7; Tosoh Bioscience, Tokyo, Japan), two immunoassays (Roche A1C and Roche Tina-quant; Roche Diagnostics), and one affinity assay (Primus Ultra-2; Primus Diagnostics, Kansas City, MO, USA). The mean value of the four HbA_1c_ measurements at the 12-week visit was used in the analysis. A detailed description of the study has been published previously^10^.

### Calculations

HbA_1c_ reflects the average glycemia over the preceding 8–12 weeks. Accordingly, the mean values of the different glycemic measures over the first three CGM measurement periods (0, 4, and 8 weeks) were used as explanatory variables and HbA_1c_ measured at the last visit (12 weeks of follow-up) was used as outcome (Supplementary Figure S1). The calculations used to derive these are specified below.

#### Preprandial glycemic measures

A measure of *pre-breakfast glycemia* was obtained from the CGM measurements at 0, 4, and 8 weeks as the average glucose concentration from *t* = −15 min to *t* = 0 min pre-breakfast. An index of *nocturnal glycemia* was calculated as mean glucose concentrations from the CGM period from 2 a.m. to 4 a.m. from the same visits.

#### Postprandial glycemic measures

From the CGM data we estimated postprandial periods as the areas under the glucose curves (AUCs) from time of intake of breakfast, lunch, and dinner and until 2 h after the meal intake. These time points were derived from the SMBG data. The mean AUC of all larger meals (breakfast, lunch, and dinner) over all measurement days at 0, 4, and 8 weeks was calculated. In addition to the AUCs, we also calculated mean *incremental AUCs* as (AUC – (preprandial glucose concentration × 2 h)) over the same measurement periods. Additionally, incremental AUCs were averaged for each meal type (breakfast, lunch, and dinner) over the three visits and further averaged over meals to provide an overall mean estimate. Peak glucose was estimated as the single highest glucose concentration obtained during one of these CGM measurement periods (0, 4, or 8 weeks).

#### Glycemic variability

As a measure of glycemic variability the mean amplitude of glycemic excursions (MAGE) was calculated from CGM data. MAGE is the mean of the differences between consecutive peaks and nadirs, only including changes >1 SD of glycemic values and thereby only capturing major fluctuations^11, 12^. Additionally, the standard deviation (SD) and coefficient of variation (CV) of the glycemic values were calculated from the CGM data. CV is the SD divided by the mean^13^. Also MAGE, SD, and CV were averaged over the three first visits (0, 4, and 8 weeks).

### Statistical analyses

Linear regression analysis was used to estimate the associations of preprandial glycemia (pre-breakfast glucose and nocturnal glucose), postprandial glycemia (total AUC, incremental AUC, and peak glucose), as well as glycemic variability (MAGE, SD, and CV) with HbA_1c_. The analyses were stratified by the three groups (no diabetes, T2D_HbA1c < 6.5%_, and T2D_HbA1c ≥ 6.5%_). Analyses were performed without adjustment and with adjustment for age, sex, BMI, and ethnicity. Prior to analysis, the eight measures of glycemia were standardized to allow direct comparisons of the strength of their association with HbA_1c_. The corresponding regression coefficients will thus reflect the difference in HbA_1c_ per 1 population SD difference in the glycemic measures.

In unadjusted linear regression analysis, using the same three groups, we calculated the proportion of variance in HbA_1c_ explained by the following categories of glycemic measures: preprandial glucose (nocturnal and pre-breakfast glucose), PPG (AUC glucose, incremental AUC glucose and peak glucose), glycemic variability (MAGE, SD, and CV), and non-glycemic factors (age, sex, BMI, and ethnicity). First, we modeled the association between HbA_1c_ and each of the four categories of glucose measures in order to estimate their individual contributions to explaining the variance in HbA_1c_. Second, the combined contribution of the four categories of glucose measures was calculated in a multiple linear regression analysis including all glycemic measures. Because the four categories of glucose measures (preprandial glucose, PPG, glycemic variability, and non-glycemic factors) to some extent are correlated, the sum of their individual contributions will likely exceed the proportions of variance explained by including all four categories in the same model. We therefore used the latter result to scale the individual contributions in order for them to add up to the total variance explained in HbA_1c_ by all the measures in combination. We also performed a sensitivity analysis, including only pre-breakfast glucose, AUC glucose, and SD as measures of preprandial and postprandial glycemia and glycemic variability, respectively.

Statistical analyses were performed in R version 3.2.3 (The R Foundation for Statistical Computing) and SAS version 9.4 (SAS Institute, Cary, NC, USA). A two-sided *P* ≤ 0.05 was used as the criterion for statistical significance in all analyses.

## Results

### Clinical characteristics

Table 1 shows the clinical characteristics of the study participants at baseline stratified by diabetes status. Compared to those with T2D, the healthy population without diabetes was younger and leaner and had a more favorable cardiometabolic profile as well as a lower use of lipid-lowering and blood pressure-lowering medications. Within the T2D population, those with the lower HbA_1c_ concentration were more likely to be men, and they were leaner and had lower systolic blood pressure than those with higher HbA_1c_ concentrations (Table 1).

**Table 1:** Characteristics of the study population by glycemic status

	No diabetes (*n* = 77)	T2D_HbA1c < 6.5%_ (*n* = 63)	T2D_HbA1c ≥ 6.5%_ (*n* = 34)	Overall *P*
*Clinical characteristics*				
Age (years)	41.1 (13.7)	56.0 (10.0)^a^	53.7 (8.8)^a^	<0.001
Male sex (%)	31.2 (21.1;42.7)	57.1 (44.0;69.5)^a^	32.4 (17.4;50.5)^b^	0.004
White ethnicity (%)	68.8 (57.3;78.9)	76.2 (63.8;86.0)	50.0 (32.4;67.6)^b^	0.034
Weight (kg)	72.3 (14.7)	89.6 (24.4)^a^	95.8 (25.5)^a^	<0.001
Waist (cm)	84.8 (13.1)	101.3 (18.0)^a^	110.7 (18.8)^a,b^	<0.001
BMI (kg/m^2^)	25.4 (4.9)	31.3 (8.2)^a^	35.2 (8.1)^a,b^	<0.001
Current smokers (%)	7.8 (2.9;16.2)	6.3 (1.8;15.5)	11.8 (3.3;27.5)	0.660
Systolic blood pressure (mm Hg)	118.7 (15.2)	128.8 (15.4)^a^	135.7 (13.8)^a,b^	<0.001
Diastolic blood pressure (mm Hg)	74.2 (9.8)	77.5 (10.4)^a^	80.8 (6.8)^a^	0.003
Total cholesterol (mmol/L)	4.7 (0.9)	4.5 (1.0)	4.2 (1.0)	0.112
HDL cholesterol (mmol/L)	1.5 (0.6)	1.2 (0.4)^a^	1.1 (0.4)^a^	<0.001
LDL cholesterol (mmol/L)	2.6 (0.9)	2.5 (1)	2.2 (0.8)	0.172
Triglycerides (mmol/L)	1.0 (0.7;1.6)	1.7 (1.1;2.7)^a^	1.7 (1.2;2.4)^a^	<0.001
Antihypertensive treatment (%)	12.0 (5.6;21.6)	54.0 (40.9;66.6)^a^	52.9 (35.1;70.2)^a^	<0.001
Lipid-lowering treatment (%)	4.0 (0.8;11.2)	52.4 (39.4;65.1)^a^	44.1 (27.2;62.1)^a^	<0.001
HbA_1c_ (%)	5.2 (0.3)	6.1 (0.6)^a^	7.5 (1.2)^a,b^	<0.001
HbA_1c_ (mmol/mol)	33.3 (2.8)	43.4 (6.3)^a^	58.1 (12.9)^a,b^	<0.001
*Preprandial glycemia*				
Pre-breakfast glucose (mmol/L)	5.8 (0.6)	7.2 (1.8)^a^	8.6 (2.8)^a,b^	<0.001
Nocturnal glucose (mmol/L)	5.7 (0.9)	6.8 (1.2)^a^	8.1 (2.6)^a,b^	<0.001
*Postprandial glycemia*				
AUC glucose (h·mmol/L)	12.1 (1.6)	15.7 (3.3)^a^	20.0 (5.2)^a,b^	<0.001
iAUC glucose (h·mmol/L)	0.5 (0.8)	1.9 (1.7)^a^	2.8 (2.5)^a,b^	<0.001
iAUC_breakfast_ glucose (h·mmol/L)	0.6 (1.2)	2.5 (2.8)^a^	4.0 (4.2)^a,b^	<0.001
iAUC_lunch_ glucose (h·mmol/L)	0.7 (1.3)	1.7 (1.9)^a^	2.5 (3.1)^a^	<0.001
iAUC_dinner_ glucose (h·mmol/L)	0.3 (1.2)	1.5 (2.5)^a^	1.6 (2.5)^a^	<0.001
Peak glucose (mmol/L)	8.9 (2.1)	12.0 (3.0)^a^	16.3 (3.8)^a,b^	<0.001
*Glycemic variability*				
MAGE (mmol/L)	1.5 (1.1;1.9)	2.8 (1.8;4.5)^a^	4.6 (3.2;6.4)^a,b^	<0.001
SD (mmol/L)	0.8 (0.6;1.0)	1.5 (1.0;2.0)^a^	2.2 (1.6;2.6)^a^	<0.001
CV (%)	13.6 (10.8;16.3)	19.8 (16.0;25.9)^a^	23.6 (18.4;30.4)^a^	<0.001

Data are shown as means (SD), medians (interquartile range), or percentages (95% CI).

Pairwise differences are illustrated by letters: ^a^ *P*  <  0.05 vs. no diabetes; ^b^ *P*  <  0.05 vs. T2D_HbA1c_  <  6.5%

Parameters with non-normally distributed values were log-transformed prior to the test.

*AUC* area under the curve for 2 h after meal intake, *iAUC* incremental area under the curve for 2 h after meal intake.

### Relationships between glycemic measures and HbA_1c_ concentrations

The median number of days with valid CGM measurements in the study population was 13 with a range from 3 to 19 days. Pairwise scatter diagrams of the interrelationships between the eight different glycemic measures by the three groups (no diabetes, T2D_HbA1c < 6.5%_, and T2D_HbA1c ≥ 6.5%_) are shown in Supplementary Figures S2–S4. In all three groups, pre-breakfast glucose was highly correlated with AUC glucose (*P* < 0.001), but not with incremental AUC glucose (*P* ≥ 0.056).

**Fig. 1:**
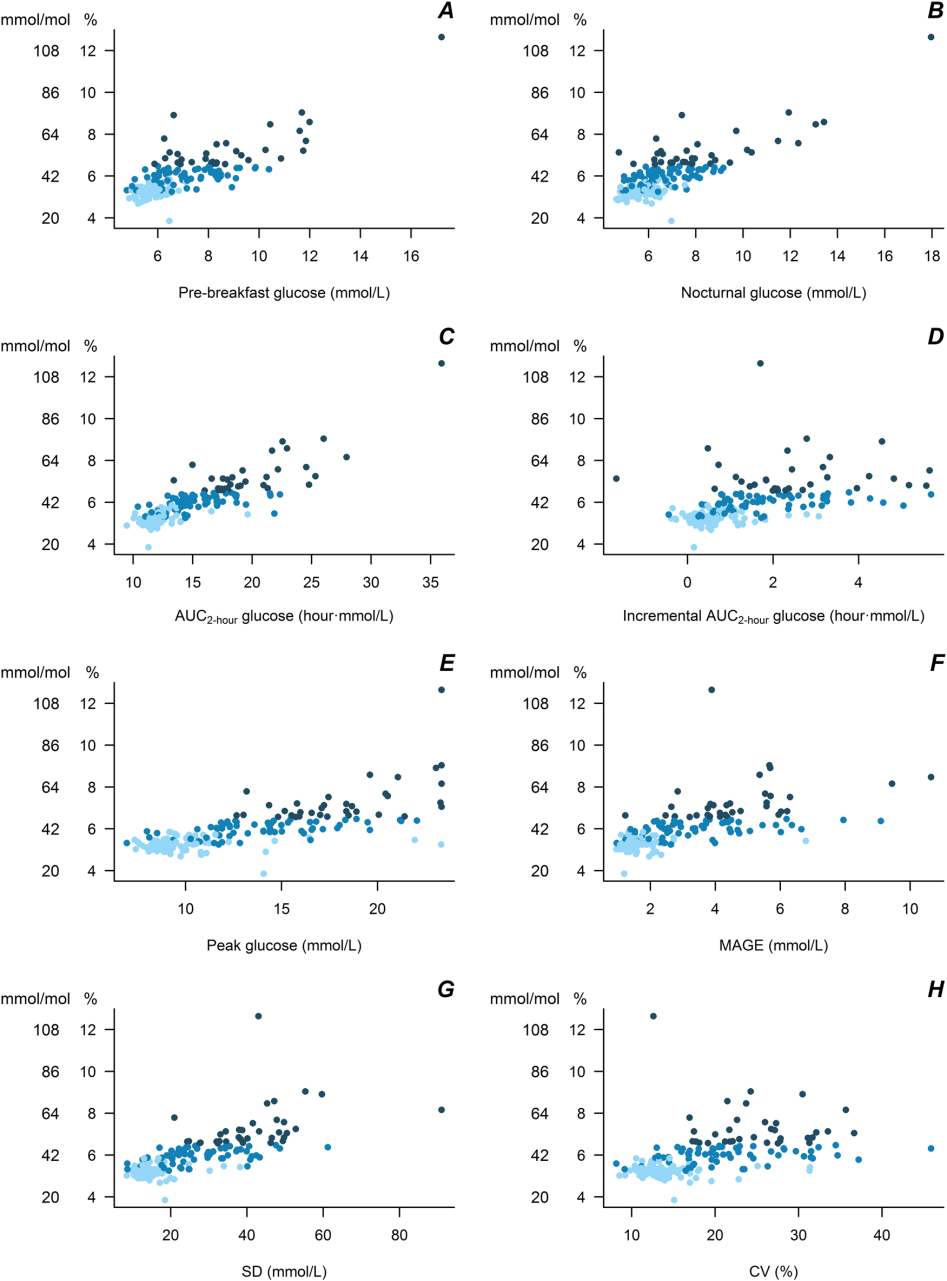
Scatter plot of HbA_1c_ measurements vs. measurements of pre-breakfast glucose (**a**), nocturnal glucose (**b**), AUC glucose (**c**), incremental AUC glucose (**d**), peak glucose (**e**), MAGE (**f**), SD (**g**), and CV (**h**) obtained from continues glucose monitoring (*P* < 0.001 for all associations). Light blue: No diabetes; blue: T2D_HbA1c < 6.5%_; dark blue: T2D_HbA1c ≥ 6.5%_

Scatter plots of HbA_1c_ measurements vs. measurements of preprandial glucose (nocturnal and pre-breakfast glucose), PPG (AUC glucose, incremental AUC glucose, and peak glucose), and glycemic variability (MAGE, SD, and CV) are shown in Fig. 1. All associations were statistically significant (*P* < 0.001), but with largest variation at higher glycemic levels.

**Fig. 2:**
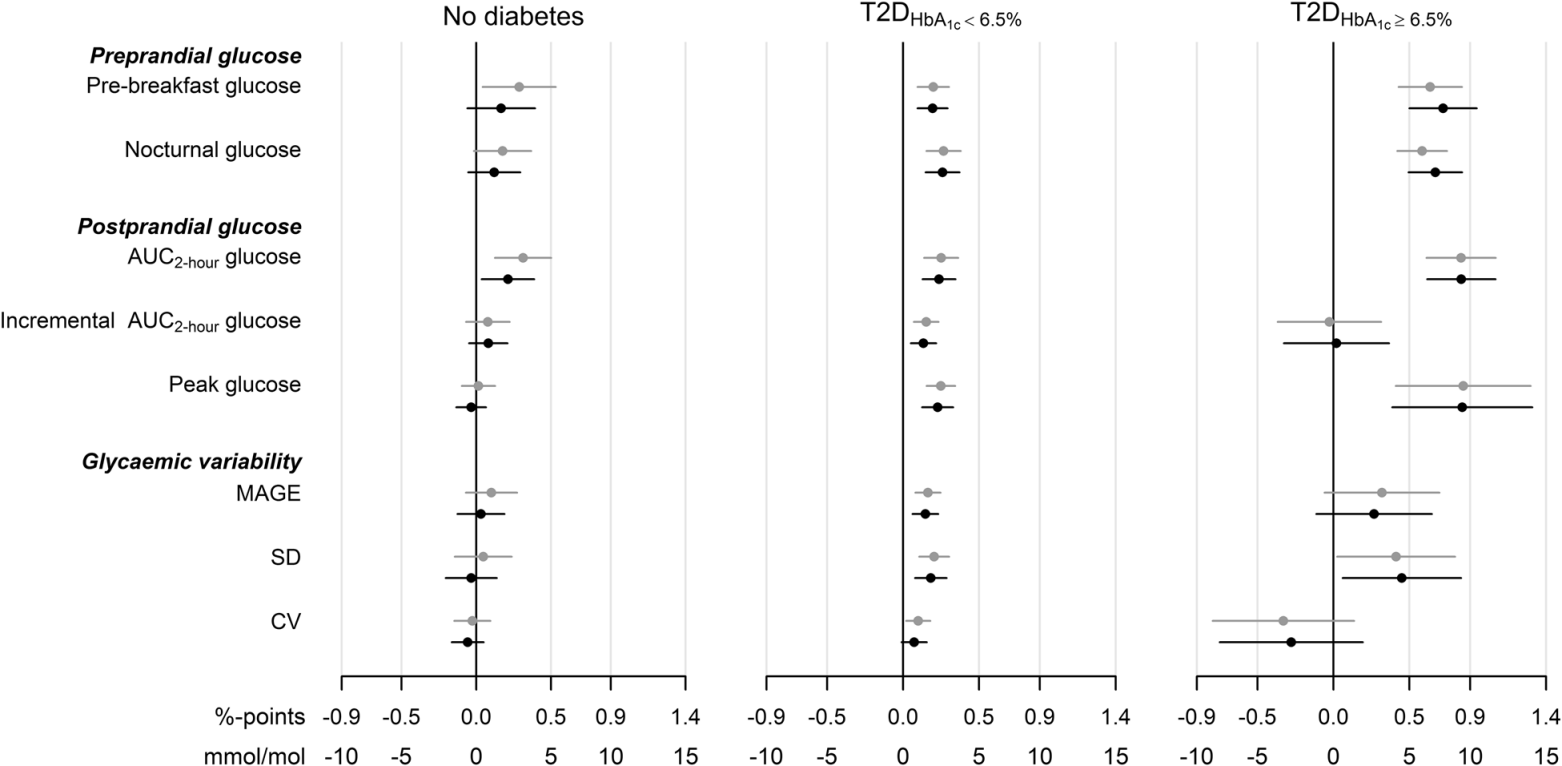
Mean (95% CI) difference in HbA_1c_ by an SD difference in glycemia for participants without diabetes, T2D_HbA1c < 6.5%_: non-insulin-treated diabetes and HbA_1c_ < 6.5%/48 mmol/mol or T2D_HbA1c ≥ 6.5%_: non-insulin-treated diabetes and HbA_1c_ ≥ 6.5%/48 mmol/mol. Estimated differences are unadjusted (gray) or adjusted for age, sex, BMI, and ethnicity (black)

Unadjusted and adjusted associations of HbA_1c_ with glycemic markers by the three groups are shown in Fig. 2. Total AUC glucose was more strongly associated with HbA_1c_ than the other glycemic markers in all three groups, and this association was robust after adjustment for confounders (Fig. 2) and for pre-breakfast glucose concentrations (*P* ≤ 0.032 for all groups).

Among individuals without diabetes, pre-breakfast glucose and AUC glucose were significantly associated with HbA_1c_ before adjustment, but in the analyses adjusted for age, sex, BMI, and ethnicity, only the association of AUC glucose with HbA_1c_ remained significant (~2 mmol/mol increase in HbA_1c_ per SD increase in AUC glucose; Fig. 2). In the T2D_HbA1c < 6.5%_ group, all the glycemic variables were significantly associated with HbA_1c_ and with largest associations for nocturnal glucose and AUC glucose—also after confounder adjustment except for CV (~3 mmol/mol increase in HbA_1c_ per SD increase in the glycemic variables; Fig. 2). For the T2D_HbA1c ≥ 6.5%_ group, the associations between glycemic variables and HbA_1c_ were overall stronger than in the other groups. For this group both pre-breakfast glucose and nocturnal glucose as well as AUC glucose, peak glucose, and SD of glucose were significantly associated with HbA_1c_ both before and after confounder adjustment and with a change in HbA_1c_ of up to 9 mmol/mol per SD increase in glycemia (Fig. 2). The incremental AUC was not associated with HbA_1c_ in the T2D_HbA1c ≥ 6.5%_ group when considering all meals or when studying breakfast, lunch, and dinner, separately (Supplementary Figure S5).

### Relative contributions of glycemic and non-glycemic factors to variation in HbA_1c_

**Fig. 3:**
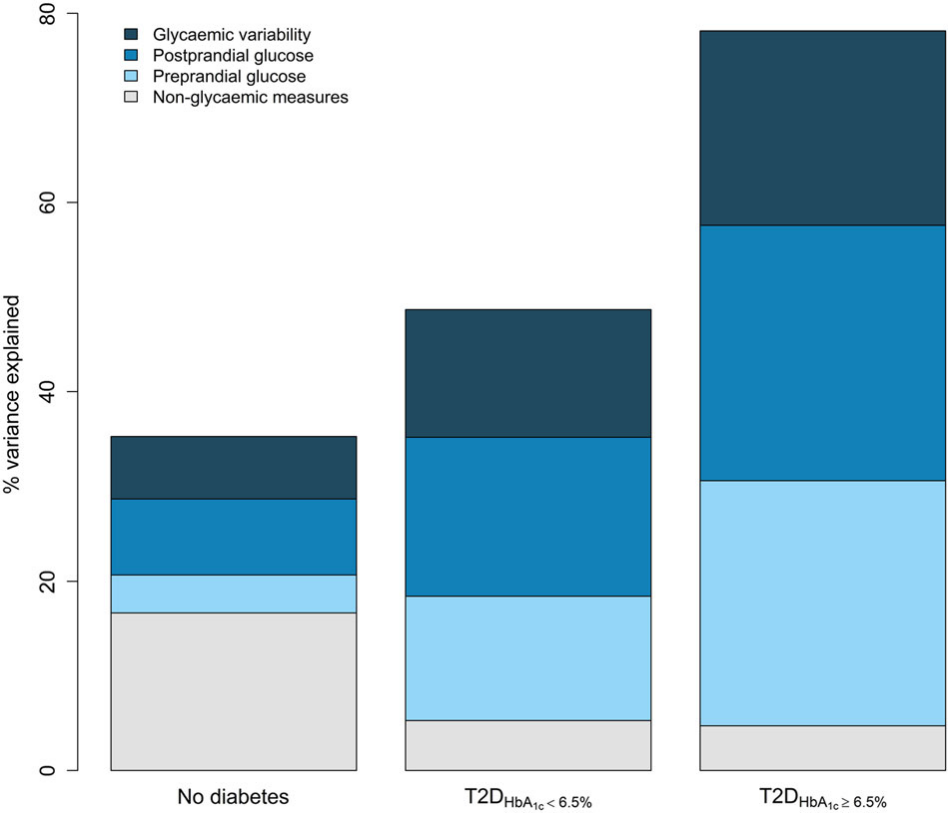
Proportion of variance explained in HbA_1c_ by non-glycemic factors (age, sex, BMI, ethnicity), preprandial glucose (pre-breakfast and nocturnal glucose), postprandial glucose (total and incremental area under the curve as well as peak glucose), and glycemic variability (MAGE, SD, and CV) by diabetes status and HbA_1c_ levels.

Figure 3 illustrates the proportion of variance in HbA_1c_ explained by preprandial glucose, PPG, glycemic variability, and non-glycemic factors in the three groups.

In the non-diabetic population, 35% of the variance in HbA_1c_ was explained by the included variables, and here the included non-glycemic factors accounted for half of the variance explained. Preprandial glucose explained 4%, PPG 8%, and glycemic variability 7% of the variance in HbA_1c_.

Among those with T2D_HbA1c < 6.5%_, 49% of the variance in HbA_1c_ was explained by the variables included in the model. Here the contribution of non-glycemic factors was small (5% of the variance explained), whereas preprandial glucose, PPG, and glycemic variability explained 13, 17, and 13%, respectively, of the variance in HbA_1c_.

For the T2D_HbA1c≥6.5%_ group, 78% of the variance in HbA_1c_ could be explained by the non-glycemic and glycemic factors included in the model. Preprandial and postprandial glycemia explained similarly large proportions (about 27% each) of the variance in HbA_1c_ and glycemic variability explained 20%. Again, non-glycemic factors only explained 5% of the variance.

Inclusion of only pre-breakfast glucose, total AUC and SD as measures of preprandial glycemic, postprandial glycemia, and glycemic variability, respectively, reduced the contributions from preprandial glucose and PPG to HbA_1c_, especially in the T2D_HbA1c < 6.5%_ group. The contribution from glycemic variability was also reduced, especially in the non-diabetic population and in the T2D_HbA1c ≥ 6.5%_ group (Supplementary Figure S6).

## Discussion

In this analysis based on repeated CGM measurements under free-living conditions, we found that PPG exposure contributed more than preprandial glucose or glucose variability exposure to variation in HbA_1c_ in non-diabetic individuals. In individuals with non-insulin-treated T2D, preprandial glucose and PPG contributed equally and slightly more than glycemic variability to variation in HbA_1c_. We also showed that glycemic factors as well as age, sex, BMI, and ethnicity accounted for only one-third of the explained variance in HbA_1c_ among individuals without diabetes, whereas these same factors accounted for nearly 80% of the explained variance in HbA_1c_ among non-insulin-treated T2D patients with HbA_1c_ ≥ 6.5%. Finally, we demonstrated that the contribution of non-glycemic factors to the explained variance in HbA_1c_ was 3–4 times higher in the non-diabetic population than in those with T2D.

The frequent CGM measurement periods during free-living conditions in combination with HbA_1c_ measured by high-quality techniques after 3 months of glucose exposure made it possible to assess the contributions of real-life glycemic exposures to the variation in HbA_1c_ in a large heterogeneous group of individuals with and without T2D. HbA_1c_ is a measure of the average blood glucose concentration over the last 8–12 weeks and is determined by glycemic as well as genetic, hematologic, and illness-related factors^1^. Changes in the lifespan of the erythrocytes can therefore affect HbA_1c_ concentrations, such that a higher mean age of erythrocytes will lead to higher HbA_1c_ concentrations^1^. The average survival of erythrocytes is slightly longer in men (117 days) than in women (106 days), so also sex can confound HbA_1c_ concentrations in a given population. Accordingly, we adjusted for age and sex in our analyses. In the ADAG study, individuals with anemia or with severe liver or renal disease were excluded from participation in order to avoid effects of iron deficiency anemia on HbA_1c_ concentrations. Factors, unrelated to age and sex, have been examined in twin studies. In non-diabetic, healthy, female twins with similar glucose tolerance, 62% of the between-person variation in HbA_1c_ was attributable to genetic factors, with the remainder reflecting age and environmental factors^14^. The relatively high contribution of non-glycemic factors to HbA_1c_ in non-diabetic individuals may explain the relatively poor overlap among fasting glucose, glucose tolerance, and HbA_1c_ in this population^15, 16^. This also suggests that markers other than HbA_1c_ might better capture the relatively subtle differences in glycemia among non-diabetic individuals^17^.

A number of observational studies have documented associations of lower dietary glycemic index and glycemic load with reduced risk of developing T2D and coronary heart disease^18, 19^. Likewise, a large trial in individuals with impaired glucose tolerance showed that administration of acarbose (a PPG lowering drug) with meals three times daily for 5 years reduced the incidence of diabetes by 18% compared to placebo^20^. Together these findings support the notion that modification of PPG by either diet or medication may have beneficial consequences for cardiometabolic health, including markers of glycemic control and thus risk of diabetes. However, the contributions to HbA_1c_ of “usual,” real-time PPG exposures has until now been unclear, because most previous studies have assessed outcomes in relation to either glycemic control in individuals or the glycemic potential of foods. Former studies consider relationships with individual variation in responses to standardized meal tests, and not under free-living conditions^8, 9^. This is an important aspect because glucose concentrations after a standard meal test mainly reflect the health status of an individual (i.e., glucose tolerance, insulin resistance, and β-cell capacity) but does not directly inform about the actual, lifestyle-related glucose exposures in the previous weeks to months. Alternatively, studies on the glycemic potential of foods or diets (e.g., glycemic index or glycemic load) consider relationships with foods or diets that are known to differ in their relative glycemic impact (as determined from standardized testing), but these also do not quantify outcomes in relation to the actual levels and variation in PPG over a sustained period of intervention or observation.

Our finding that PPG, as estimated by the total AUC for glucose following meals, seems to be the main contributor to HbA_1c_ in non-diabetic individuals provides a link between PPG and HbA_1c_ that supports the notion that lower PPG is beneficial in terms of preventing future diabetes and reducing risk for cardiovascular disease^4, 18, 19^. However, in our study population each SD higher AUC was associated with a 0.2%-point (2 mmol/mol) higher HbA_1c_, indicating that the magnitude of the association was rather modest.

Another aspect of PPG is glycemic variability, which was associated with HbA_1c_ in individuals with non-insulin-treated T2D, but not in individuals without diabetes. In the non-diabetic population mean MAGE was 1.5, which is 2–3 times lower than in the diabetes population. Other studies have estimated MAGE to be between 1.6 and 1.8 in non-diabetic individuals^21–23^, underscoring that the non-diabetic individuals included in the ADAG study are healthier than the general non-diabetic population.

Overall, we found that preprandial and PPG explained an equal amount of variance in HbA_1c_ in individuals with T2D. Furthermore, glycemic features explained a much larger proportion of the variation in those with T2D than in the non-diabetic population. Other studies using CGM for assessment of glycemia under free-living conditions have also found equal contributions of fasting and postprandial hyperglycemia to HbA_1c_ in patients with T2D, but with a tendency of a greater contribution of PPG at lower HbA_1c_ concentrations and higher contribution of fasting hyperglycemia at higher HbA_1c_ concentrations^24–26^. The latter result is in accordance with the findings by Monnier et al.^9^ who suggested that the relative contribution of PPG decreased, whereas the relative contribution of fasting glucose increased, from the lowest to the highest HbA_1c_ quintile among non-insulin-treated T2D patients with HbA_1c_ ranging from ~6 to 12% (42–108 mmol/mol). The use of CGM to measure real-life exposures in the entire study population and the exclusion of individuals treated with insulin in our study are likely to explain the differences between our results and the results by Monnier et al^9^.

A limitation of our study was the observational design, which limited the possibility to study whether differences in PPG exposures were caused by lifestyle behaviors or use of oral glucose-lowering medications (in T2D only) during the measurement periods or whether they were due to pre-existing glucose intolerance/insulin resistance. We used “pre-breakfast” glucose concentrations as baseline in the calculation of the incremental AUC, which appeared higher than the fasting glucose concentrations measured during screening. Accordingly, the true PPG exposures may have been underestimated and the pre-breakfast exposures overestimated in our study. Another limitation was that information on physical activity and dietary intake was not collected concomitant with the CGM periods. Such information would be relevant to collect in future studies, particularly in individuals without diabetes, in order to understand how different foods, eating patterns, or exercise bouts as well as timing of eating affect daily blood glucose concentrations, peak glucose, and overall glycemia.

In conclusion, we found that PPG exposure contributed more than preprandial glucose or glucose variability to variation in HbA_1c_ in non-diabetic individuals, whereas preprandial glycemia and postprandial glycemia contributed equally to the variation in HbA_1c_ in individuals with T2D. We could only explain one-third of the variance in HbA_1c_ by glycemic factors, age, sex, BMI, and ethnicity among individuals without diabetes, whereas these factors explained nearly 80% of the variance in HbA_1c_ among non-insulin-treated T2D patients with HbA_1c_ concentrations ≥6.5%. Knowledge from this study can be useful to predict clinically meaningful effects on HbA_1c_ of dietary interventions targeting PPG in non-diabetic and non-insulin-treated individuals with T2D with a wide range of glycemic control.

## Electronic supplementary material

Suppl. information: Figure legends

Suppl. Figure 1

Suppl. Figure 2

Suppl. Figure 3

Suppl. Figure 4

Suppl. Figure 5

Suppl. Figure 6
