# Supplementary material for: Relative contributions of preprandial and postprandial glucose exposures, glycemic variability, and non-glycemic factors to HbA1c in individuals with and without diabetes
Source: Nutr Diabetes. 2018 Jun 1;8:38. doi: 10.1038/s41387-018-0047-8 (PMC5981454; doi:10.1038/s41387-018-0047-8)
Supplement: Supplementary file 1 — Suppl. information: Figure legends [file 41387_2018_47_MOESM1_ESM.docx]

**Supplementary information**

**Supplementary Figure S1:** Flow chart of number of study participants (N) and observations (n) at the different visits.

**Supplementary Figure S2:** Pairs plots of the glycemic measures in healthy individuals.

**Supplementary Figure S3:** Pairs plots of the glycemic measures in non-insulin treated diabetes and HbA_1c_ < 6.5%/48 mmol/mol (T2D_HbA1c<6.5%_).

**Supplementary Figure S4:** Pairs plots of the glycemic measures in non-insulin treated diabetes and HbA_1c_ ≥ 6.5%/48 mmol/mol (T2D_HbA1c≥6.5%_).

**Supplementary Figure S5:** Difference in HbA_1c_ by a SD difference in incremental AUC glucose for all meals, breakfast, lunch and dinner for participants without diabetes, T2D_HbA1c<6.5%_**:** non-insulin treated diabetes and HbA_1c_ < 6.5%/48 mmol/mol or T2D_HbA1c≥6.5%_**:** non-insulin treated diabetes and HbA_1c_ ≥ 6.5%/48 mmol/mol. Estimated differences are unadjusted (grey) or adjusted for age, sex, BMI and ethnicity (black).

**Supplementary Figure S6:** Proportion of variance explained in HbA_1c_ by non-glycemic measures (age, sex, BMI, ethnicity), pre-breakfast glucose, area under the curve (AUC_2-hour_) for glucose, and standard deviation (SD) of glucose values by diabetes status and HbA_1c_ levels.
