# Supplementary figures and images for: Relative contributions of preprandial and postprandial glucose exposures, glycemic variability, and non-glycemic factors to HbA1c in individuals with and without diabetes
Source: Nutr Diabetes. 2018 Jun 1;8:38. doi: 10.1038/s41387-018-0047-8 (PMC5981454; doi:10.1038/s41387-018-0047-8)

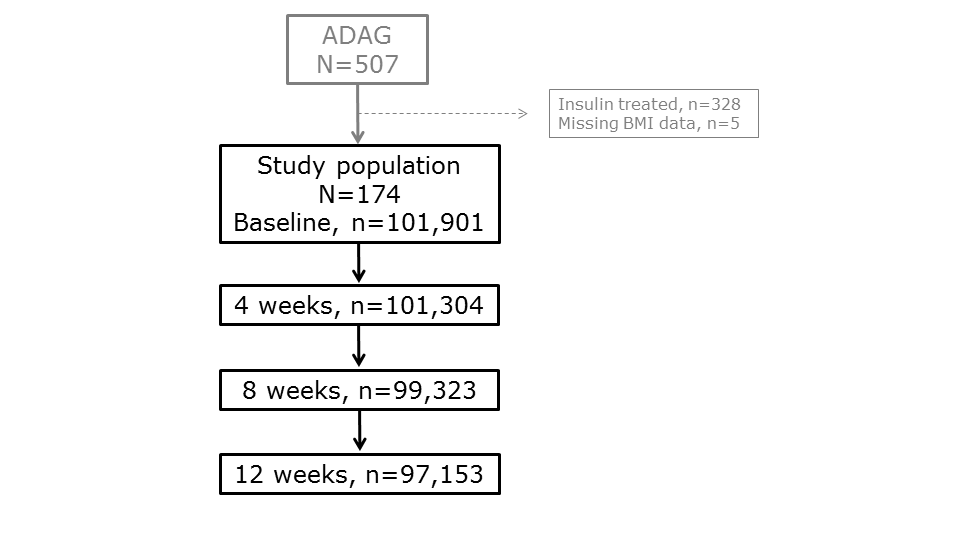

Supplement: Supplementary file 2 — Suppl. Figure 1 [file 41387_2018_47_MOESM2_ESM.tif]

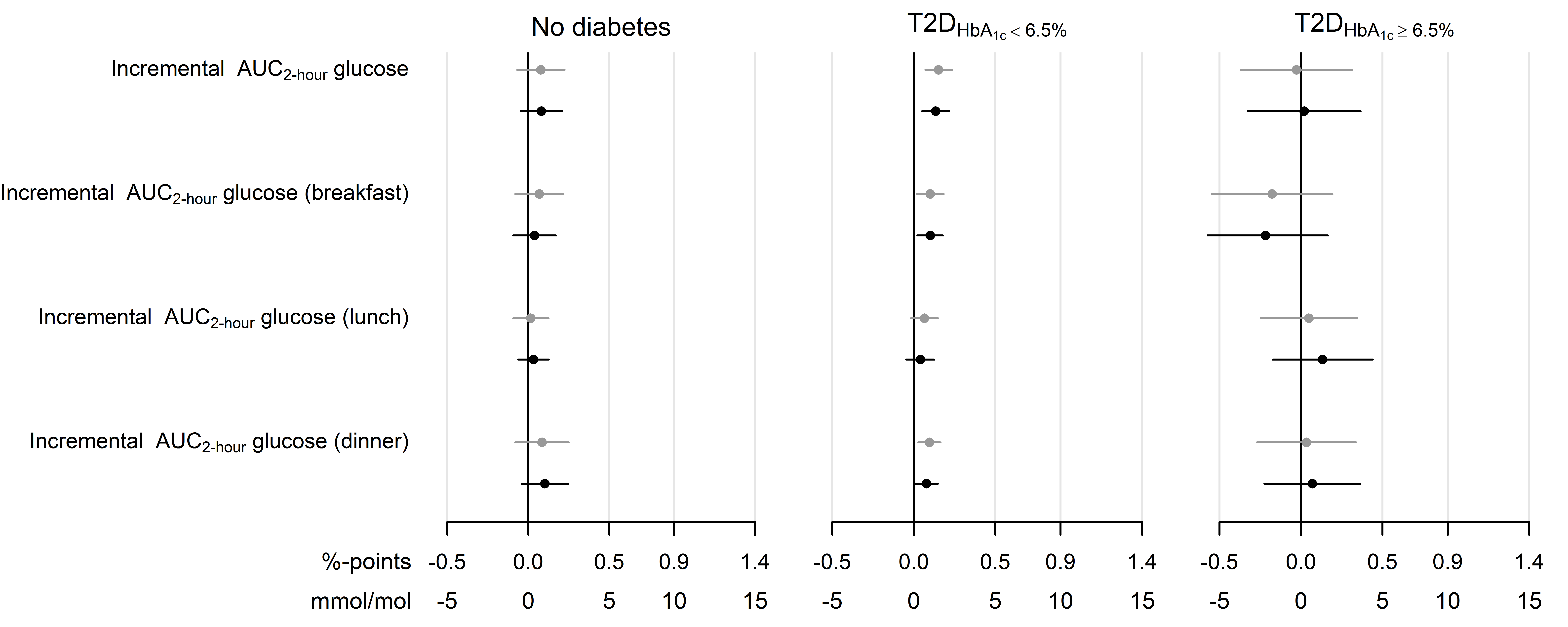

Supplement: Supplementary file 6 — Suppl. Figure 5 [file 41387_2018_47_MOESM6_ESM.tif]
